# Functional hypergraph uncovers novel covariant structures over neurodevelopment

**DOI:** 10.1002/hbm.23631

**Published:** 2017-05-11

**Authors:** Shi Gu, Muzhi Yang, John D. Medaglia, Ruben C. Gur, Raquel E. Gur, Theodore D. Satterthwaite, Danielle S. Bassett

**Affiliations:** ^1^ Department of Psychiatry University of Pennsylvania Philadelphia Pennsylvania; ^2^ Applied Mathematics and Computational Science Graduate Group University of Pennsylvania Philadelphia Pennsylvania; ^3^ Moss Rehabilitation Research Institute Elkins Park Pennsylvania; ^4^ Department of Electrical and Systems Engineering University of Pennsylvania Philadelphia Pennsylvania; ^5^ Department of Bioengineering University of Pennsylvania Philadelphia Pennsylvania

**Keywords:** brain network, core–periphery, functional connectivity, network modules

## Abstract

Brain development during adolescence is marked by substantial changes in brain structure and function, leading to a stable network topology in adulthood. However, most prior work has examined the data through the lens of brain areas connected to one another in large‐scale functional networks. Here, we apply a recently developed hypergraph approach that treats network connections (edges) rather than brain regions as the unit of interest, allowing us to describe functional network topology from a fundamentally different perspective. Capitalizing on a sample of 780 youth imaged as part of the Philadelphia Neurodevelopmental Cohort, this hypergraph representation of resting‐state functional MRI data reveals three distinct classes of subnetworks (hyperedges): clusters, bridges, and stars, which respectively represent homogeneously connected, bipartite, and focal architectures. Cluster hyperedges show a strong resemblance to previously‐described functional modules of the brain including somatomotor, visual, default mode, and salience systems. In contrast, star hyperedges represent highly localized subnetworks centered on a small set of regions, and are distributed across the entire cortex. Finally, bridge hyperedges link clusters and stars in a core–periphery organization. Notably, developmental changes within hyperedges are ordered in a similar core–periphery fashion, with the greatest developmental effects occurring in networked hyperedges within the functional core. Taken together, these results reveal a novel decomposition of the network organization of human brain, and further provide a new perspective on the role of local structures that emerge across neurodevelopment. *Hum Brain Mapp 38:3823–3835, 2017*. © **2017 The Authors. Human Brain Mapping Published by Wiley Periodicals, Inc.**

## INTRODUCTION

Understanding the human brain as a networked system has offered important insights into the development of brain function across the lifespan [Bassett and Sporns, 2017). In this perspective, brain regions are treated as network nodes, and functional connections between brain regions are treated as network edges [Bullmore and Bassett, 2011]. Studies examining functional brain networks at rest measured with functional MRI have revealed a modular functional organization [Dosenbach et al., 2007; Power et al., 2011] comprised of reproducible network communities such as the default‐mode [Raichle et al., 2001], cognitive control [Sridharan et al., 2008], salience [Seeley et al., 2007], visual [Lowe et al., 1998], and somatomotor [Biswal et al., 1995] systems. These modules evolve considerably during development in adolescence [Power et al., 2010; Satterthwaite et al., 2013b; Gu et al., 2015a], and are thought to allow for the expansion of cognitive and behavioral capabilities that defines this period.

While such studies have offered critical insights into the network neurophysiology of development, they constitute relatively coarse levels of interrogation. Modules are mesoscale structures, defined as sets of brain areas. As the module structure depends on the average properties of network edges, they are relatively insensitive to how these edges are combined with each other. To resolve this level of detail, we take an alternative approach by treating the network edge as the unit of interest [Bassett et al., 2014; Davison et al., 2015, 2016]. This choice is guided by the fact that edges may develop differentially in a coordinated fashion over the lifespan [Davison et al., 2016], leading to architectural features that cannot simply be characterized by modules or the nodes that compose them [Bassett et al., 2014]. Intuitively, such developmental coordination of functional connections may be driven by intrinsic computations [Bassett et al., 2014], and subsequently have mutually trophic effects on underlying structural connectivity [Bassett et al., 2008]. From a computational standpoint, co‐varying functional connections can be thought of as circuits—edges that link disparate computational units—that may form more fundamental structures that prefigure the emergence of well‐described cognitive systems observed in adulthood.

To investigate functional brain network architecture at this finer scale, we examine high‐resolution edge‐based hypergraphs in a large sample of youth imaged as part of the Philadelphia Neurodevelopmental Cohort. In contrast to typical functional networks where nodes represent brain regions, hypergraphs are built on the pairwise correlations between network edges across individuals, allowing for the detection of groups of coherent edges known as hyperedges. This particular emphasis on functional edges enables us to address several specific hypotheses. First, we expected that hypergraphs would corroborate and extend findings from prior (region‐based) network analyses, and reveal cluster hyperedges that form densely interconnected brain systems. Second, we hypothesized that this approach would allow us to uncover novel types of subgraphs that were distinguishable from traditional network modules. Third and finally, we hypothesized that age‐related change of hyperedge strength during adolescence would differ by hyperedge type. As described below, such edge‐base analyses allowed us to uncover novel fine‐scale functional subgraphs that display differential patterns of development during adolescence.

## MATERIALS AND METHODS

### Data Acquisition and Preprocessing

Data were acquired as part of a collaboration between the Center for Applied Genomics (CAG) at Children's Hospital of Philadelphia (CHOP) and the Brain Behavior Laboratory at the University of Pennsylvania (Penn). Study procedures were reviewed and approved by the Institutional Review Board of both CHOP and Penn. Resting‐state fMRI data considered in the present study consisted of 780 healthy youth age 8–22 years. For full details regarding sample construction, inclusion, and exclusion criteria see Satterthwaite et al. [2013]. Of these youth, 333 were male and 447 were female. For a thorough account of cognitive performance in the PNC, see Gur et al. [2012] and Moore et al. [2015].

All subject data were acquired on the same scanner (Siemens Tim Trio 3 Tesla, Erlangen, Germany; 32 channel head coil) using the same imaging sequences. Blood oxygen level‐dependent (BOLD) fMRI was acquired using a whole‐brain, single‐shot, multislice, gradient‐echo (GE) echoplanar (EPI) sequence of 124 volumes (372 s) with the following parameters TR/TE = 3000/32 ms, flip = 90°, FOV = 192 × 192 mm, matrix = 64 × 64, slice thickness/gap =3 mm/0 mm. The resulting nominal voxel size was 3.0 × 3.0 × 3.0 mm. A fixation cross was displayed as images were acquired. Subjects were instructed to stay awake, keep their eyes open, fixate on the displayed crosshair, and remain still.

Functional imaging used tools from FSL (FMRIB's Software Library) and AFNI with a preprocessing scheme described elsewhere [Satterthwaite et al., 2013a, 2014b]. A detailed description of the preprocessing pipeline specifically as applied to this data can be found in Supporting Information. The data reported in this article have been deposited in the database of Genotypes and Phenotypes (dbGaP), http://www.ncbi.nlm.nih.gov/gap (accession no. phs000607.v1.p1).

### Functional Network Construction

We extracted regional mean BOLD time series from 264 functionally defined regions (each region constituting a 5 mm sphere) covering cortical and subcortical areas [Power et al., 2011]. The Power parcellation has important merits, including demonstrating a superior reliability of fMRI‐based graph theoretical properties during working memory, emotion processing, and resting state [Cao et al., 2014]. Second, the Power parcellation has previously been used in the PNC data set [Satterthwaite et al., 2013a, 2013b; Gu et al., 2015a; Chai et al., 2017]. By maintaining consistency with these other studies, we facilitate comparison across analysis methods and data types.

Consistent with prior work [Bassett et al., 2011b, 2013b], we estimated functional connectivity between any two pairs of regions using a wavelet coherence [Grinsted et al., 2004] in the frequency interval of approximately 0.01–0.08 Hz. We chose to use a wavelet coherence for several statistical reasons. First, we note that wavelet‐based methods for decomposing the preprocessed fMRI time series offer useful denoising properties [Fadili and Bullmore, 2004] are robust to outliers [Achard et al., 2006], and can be used to construct useful null models [Pritchard et al., 2014]. Wavelet‐based methods extract frequency‐specific information in the time series without the edge effects characteristic of bandpass filtering [Percival and Walden, 2000]. The benefits of wavelet‐based decompositions are particularly relevant to fMRI time series because of their long memory nature, displaying a positive and slowly decaying autocorrelation function [Maxim et al., 2005; Wink et al., 2006]. Traditional time‐ and frequency‐domain measures of association (including correlation) are not properly estimable for long memory time series [Beran, 1994]. In contrast, wavelets provide a means of reliably estimating correlation between long memory time series [Whitcher et al., 2000; Gencay et al., 2001], including those derived from resting‐state fMRI data [Achard & Bullmore, 2007; Bullmore et al., 2004; Achard et al., 2008].

Second, we chose to use an estimate of coherence because a simple linear Pearson correlation is sensitive to outliers [Devlin et al., 1975; Huber, 2004]—such as those caused by motion artifact—and moreover provides a perhaps overly strict and narrow measurement of functional connectivity based on only pointwise differences between two signals [Gayen, 1951]. Coherence, in contrast, provides a broader estimate of associations between time series [White and Boashash, 1990], being sensitive to statistical similarities in the power spectra of the two BOLD activity traces. It is given by a ratio of the cross‐spectral density between time series *x* and time series *y*, and the product of the autospectral density of *x* and the autospectral density of *y*. In the context of fMRI time series analysis, evidence suggests that the coherence is a useful measure of the magnitudes of time series similarity that is independent of inter‐regional differences in the HRF, which can cause nontrivial variations in a Pearson correlation coefficient that are independent of the underlying neural activity [Sun et al., 2004, 2007]. Moreover, coherence as a measure of functional connectivity has proven particularly helpful in the examination of functional neuroimaging data from a network perspective [Bassett et al., 2011a, 2011b; Chai et al., 2016; Telesford et al., 2016; Gu et al., 2015b].

The fully weighted adjacency matrix therefore represents the functional brain network for a given subject in which network nodes represent brain regions and network edges represent functional connections between those regions [Zhang et al., 2016].

### Hypergraph Construction

To construct an edge‐by‐edge hypergraph [Berge and Minieka, 1973], we stacked subject adjacency matrices to create a three‐dimensional adjacency tensor with elements $A_{ijs}$, where *s* indexes over subjects [Bassett et al., 2014]. For an edge connecting a given pair of regions i and j, the elements $A_{ijs}$, for all s could be treated as a vector: a series of observations over the entire sample (*N* = 780). For every pair of edges, $A_{ijs}$, and $A_{kls}$ for all s, we computed the Pearson correlation coefficient between these edge weight vectors $H_{mn}$, where m indexes over edge pairs i and j and *n* indexes over edge pairs *k* and *l*, and we stored these values in the E × E hypergraph H. Following Bassett et al. [2014] and to control the false‐positive rate, we thresholded the matrix H by setting all correlation coefficient values $H_{mn}$ to zero whose respective *P* values were greater than 0.05. Intuitively, the hypergraph H provides a cross‐sectional representation of functional connections that co‐vary with one another. Entries are positive if the weights of the corresponding edges are positively correlated over subjects, and entries are negative if the weights of the corresponding edges are negatively correlated over subjects. We note that 4.57% of entries were negative, and for simplicity in the following analyses, we set these values to zero.

### Hyperedge Archetypes

We identified hyperedges—significant clusters of co‐varying edges—by applying a common network‐based community detection algorithm [Porter et al., 2009; Fortunato, 2010] to the hypergraph H. Specifically, we applied a generalized version of a Louvain‐like locally greedy algorithm [Blondel et al., 2008] to maximize a modularity quality function [Newman, 2004]. This widely used algorithm is computationally efficient even for large networks, and is publically available at http://netwiki.amath.unc.edu/GenLouvain/GenLouvain.

Next, we defined hyperedge archetypes that each displayed interpretable spatial configurations in the brain, and that corresponded to differential roles in the dynamic processes of integration and segregation. For each hyperedge, we listed the edges that composed the associated cluster *r*, and then determined the set of nodes (brain regions) that were touched by at least one of those edges. We then defined a binary adjacency matrix whose elements indicated the presence (1) or absence (0) of an edge between nodes *i* and *j* that were also present in the cluster *r*. We observed that the matrices $B^{r}$ fell into one of three categories: focal star hyperedge, bridging bipartite hyperedge, and cluster hyperedge (Fig. 2). The classification here follows a two‐step procedure: (i) if the graph cannot be statistically partitioned into a bipartite structure, then it is defined as a *cluster*; (ii) if the graph can be statistically partitioned into a bipartite structure, then we check the number of nodes in the smaller of the two parts; if the number is less than 4, then the graph is defined as a *star*; otherwise, it is defined as a *bridge*. Thus, intuitively, stars consist of edges that are linked to one another via one, two, or three nodes. Bridges consist of edges that connect one set of nodes to a second set of nodes, but do not connect nodes within the same set. Clusters consist of edges that connect nodes both within and between sets. See Supporting Information for a detailed description of the clustering method and Figure S4 for details on parameter choices.

### Hyperedge Connector Estimation

Bridges are particularly interesting as they represent the collection of edges that can connect two or more hyperedges with one another. We hypothesized that the bridges connected clusters to stars. To test our hypothesis, we performed the following analysis: First, we set the valid stars and the clusters as fundamental modules. Second, for each valid bridge, we computed the number of overlap regions on each side with every fundamental module and calculated the *P* values versus the null distribution where each side of the bridge is randomly chosen from among all possible regions while preserving size. Third, we thresholded the *P* values in Step 2 by applying a Benjamini–Hochberg procedure to control the false‐discovery rate (FDR) at *Q* < 0.05. Finally, according to the type of module on each end of the bridge, we classified the bridge into three subtypes of connectors: star–star connector, star–cluster connector, and cluster–cluster connector, and we examined how many significant bridges there were of each subtype, normalized by the number of possible bridges of that subtype.

### Edge Correlation Comparison

For the edge comparison in Figure 3, we computed the correlation among edges within each predefined module and compared its distribution with that of the average correlation of edge pairs within the stars centered within the same module. Multiple comparisons were controlled for using FDR (*Q* < 0.05) [Storey, 2002].

### Linear Model of Developmental Effects

To test which cluster hyperedges displayed associations with age, we mapped back the 6 clusters to each subject, computed the average connection strength and investigated associations with age while co‐varying for in‐scanner motion.

See Supporting Information for additional methodological details.

## RESULTS

**Figure 1:**
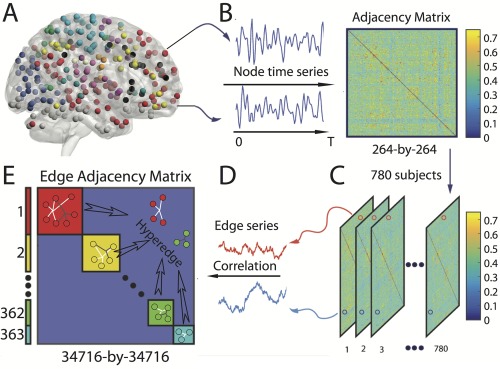
Schematic of hypergraph construction. (A) We first extract time series for each region of interest. (B) Next, we calculate the functional connectivity between pairs of regions using a wavelet‐based coherence, yielding an adjacency matrix. We perform the steps outlined in panels (A) and (B) for each of the 780 youth in the Philadelphia Neurodevelopmental Cohort and (C) stacked the adjacency matrices across subjects. (D) We extract the vector of weights for each edge over subjects. (E) Finally, we generate an edge‐by‐edge adjacency matrix (or hypergraph) [Bassett et al., 2014] by computing the Pearson correlation coefficient between pairs of edge weight time series. A hyperedge is then defined as a cluster of edges that co‐vary with one another; we can identify these clusters by applying community detection techniques to the hypergraph. [Color figure can be viewed at http://wileyonlinelibrary.com]

We constructed high‐resolution hypergraphs (Fig. 1) using resting‐state fMRI data acquired from 780 youth between the ages of 8 and 22 years [Satterthwaite et al., 2014]. For each participant, we created adjacency matrices by calculating the wavelet coherence between all 34,716 pairs of 264 functionally defined regions. Adjacency matrices were concatenated across subjects to create a three‐dimensional matrix, and then collapsed to an edge‐by‐edge matrix whose elements were given by the Pearson correlation coefficient between each pair of edges. This hypergraph of dimensions 34,716 × 34,716 summarized the degree to which functional connections co‐varied with one another over subjects.

**Figure 2:**
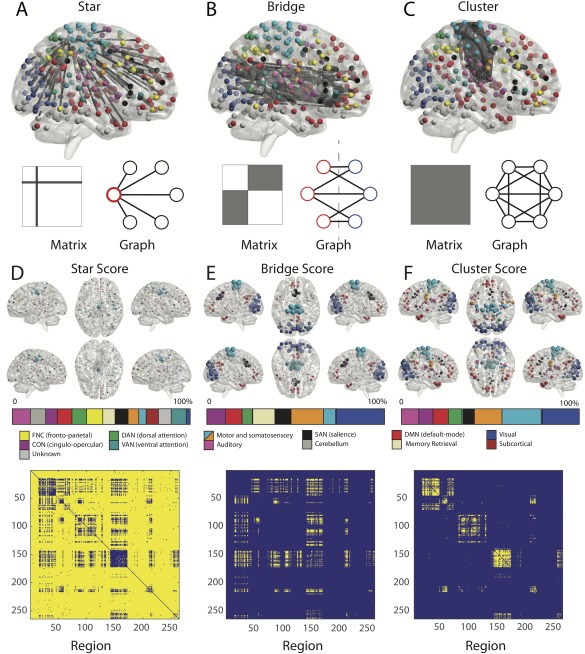
Hyperedge archetypes. Example of star (A), bridge (B), and cluster (C) hyperedges with accompanying graph representations. In the 363 hyperedges, we observed 326 that displayed star‐like structure (stars), 31 that displayed bipartite‐like structure (bridges), and 6 that displayed network‐like structure (clusters). We define (D) a star score as the number of times a node acts as a core within a star‐like structure, (E) a bridge score as the number of edges belonging to a bipartite hyperedge that emanated from a given node, and (F) a cluster score as the number of edges belonging to a cluster hyperedge that emanated from a given node. The color bars display the percentage of the weighted scores in each system normalized by the number of regions in the system. [Color figure can be viewed at http://wileyonlinelibrary.com]

**Figure 3:**
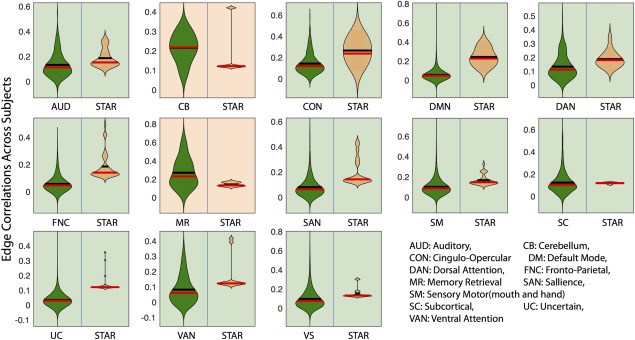
Edge correlations across subjects. To investigate whether the star hyperedges displayed higher cohesiveness among edges than well‐known cognitive systems or network modules, we compared the star hyperedges and 13 predefined modules [Power et al., 2011] with the null hypothesis that the pairwise similarity of edges in star hyperedges was no higher than that of the edges within the modules. Except for the two smallest systems (the cerebellum and memory retrieval), all other null hypotheses were rejected with the FDR‐corrected q<0.001. Results emphasize the higher level of coherence present in star hyperedges than in traditional node‐based network modules. [Color figure can be viewed at http://wileyonlinelibrary.com]

### Hyperedges Reveal Novel Architectural Motifs

Within the full hypergraph, we identified statistically significant functional hyperedges: groups of edges that co‐varied in strength over subjects [Bassett et al., 2014]. In the 363 significant hyperedges (Fig. 1E), we detected 3 distinct topological classes (Fig. 2): *stars* (326 of 363), *bridges* (31 of 363), and *clusters* (6 of 363). Stars were the most numerous hyperedges in the hyergraph. In mathematics, a star graph is one in which edges emanate from a small set of nodes (Fig. 2A). These star‐like structures indicate the presence of neurophysiological drivers of functional connectivity that are localized to very few ( ≤3) brain regions. Bridges are bipartite graphs that are composed of edges connecting two separate sets of nodes (Fig. 2B). These bridging structures suggest the existence of connectors among hyperedges. Clusters include edges that densely link spatially distributed regions (Fig. 2C).

### The Three Archetypes are Distributed Differently in Space

We expected that bridges would be particularly prevalent in functional modules where clusters were also present (i.e., visual, motor, default mode, and cingulo‐opercular systems). In contrast, we expected that peripheral stars would be likely to be more present over the entire cortex. We quantified the loading of the three types of hyperedges onto individual brain regions by counting nodal occupation of the hyperedges. The presence of cluster hyperedges was quantified by a cluster score, which summarized how many times the nodes participated in the significant cluster hyperedges. The presence of bridge hyperedges was quantified similarly, and we refer to these estimates as the bridge scores. As indicated in Figure 2E, the bridge score was elevated in known functional systems where cluster hyperedges were also found (Fig. 2F), including the visual system, somatomotor system, default mode network, and salience systems. A co‐participation plot emphasized that bridge hyperedges exclusively formed links between these clusters to other nodes outside the clusters (Fig. 2E bottom). The presence of stars was quantified by a star score, which summarizes the degree to which a given region participates in a star hyperedge. In contrast to the striking regional focus of both cluster and bridge hyperedges, star hyperedges were widely distributed across the cortex (Fig. 2D).

### Stars Imply More Cohesive Collections of Edges Than Predefined Cognitive Systems

As we note in the previous section, stars were broadly distributed across the brain (Fig. 2). To quantify this observation, we examined a set of systems or modules defined *a priori* [Power et al., 2011] and tested the null hypothesis that the cross‐subject Pearson's correlation of edge weights of stars centered in nodes within a module was higher than edge weights within the module overall. This would establish whether stars are fundamentally more cohesive subunits than previously detected major systems. The anticipated result of this test was not clear *a priori* for two reasons: (i) edges included in the hypergraph were required to be stronger than *r* = 0.07, and therefore, these edges need not be particularly high in connectivity, and (ii) stars tended to contain between‐system connections and therefore need not be as strong as within‐system connections. This approach revealed that stars were more coherent than all cognitive systems (FDR‐corrected for multiple comparisons, Q < 0.001), with the exception of the cerebellum (composed of 4 nodes) and memory retrieval systems (composed of 5 nodes). In these latter two systems, due to their small module size, both the cohesiveness of modules and the cohesiveness of stars may vary greatly, widening the null distribution and leading to a nonsignificant result. Yet the significant findings in all other cognitive systems underscore the utility of the hypergraph approach in uncovering more coherent substructures than traditional community detections techniques uncovering network modules. It also suggests that star‐shaped hyperedges may constitute one of the fundamental units of the brain's functional architecture.

### The Functional Core of Cluster Hyperedges

**Figure 4:**
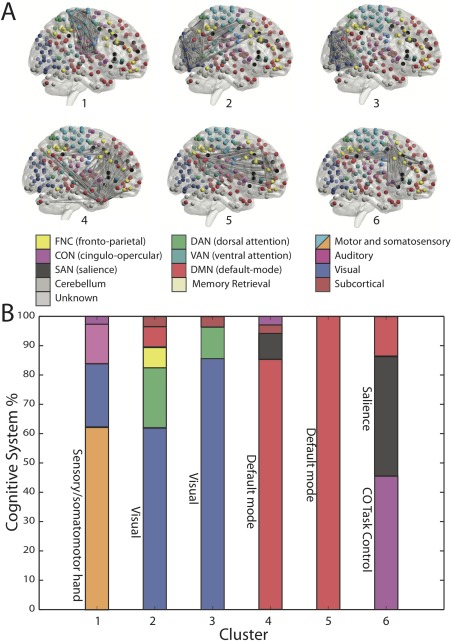
Cluster hyperedges. (A) Six of the 363 hyperedges were clusters. Each cluster displayed a distinct spatial organization that recapitulated well‐known functional systems. (B) Each cluster hyperedge connects a set of nodes, and each node (brain region) belongs to a previously defined cognitive system. Of the nodes present in a cluster, we show the percentage that is a part of each cognitive system. [Color figure can be viewed at http://wileyonlinelibrary.com]

We next turned to examining the nature of the cluster hyperedges, which occupy a central role in the hypergraph architecture. We observed that (Fig. 4) clusters are remarkably similar to known functional subnetworks [Power et al., 2011]. Of the six clusters identified, two were predominantly composed of regions in the default mode, two were predominantly composed of regions in visual cortex, one was largely composed of areas in somatosensory cortex, and one was largely composed of areas in the salience and cingular‐opercular task control systems. These results demonstrate that cluster hyperedges recapitulate previously described large‐scale functional networks that have strongly coherent, dense connections.

### Bridges Connect Core Clusters and Peripheral Stars

**Figure 5:**
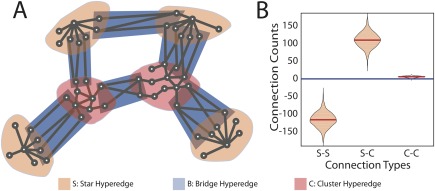
Bridges connect stars to clusters. (A) A schematic figure shows bridge hyperedges (blue) connect stars (peach) and clusters (pink). (B) Testing the intersection of hyperedges versus the null distribution where the nodal occupation is uniformly sampled from all brain regions (see Methods), we demonstrate that bridges are more likely to connect stars with clusters than expected ( $P<1\times 10^{-20}$). Moreover, bridges are less likely to connect stars to other stars ( $P\;<\;1\times 10^{-20}$). [Color figure can be viewed at http://wileyonlinelibrary.com]

Intuitively, bridges are groups of edges that can facilitate network integration by linking two distinct sets of brain areas (Fig. 5A). Given that hyperedges neatly composed 3 distinct categories, we hypothesized that bridge hyperedges served to link the densely connected core of cluster hyperedges and less‐connected star hyperedges. More specifically, the null hypothesis here was that bridges randomly connected two parts of the brain and were not significantly enriched for any of the following: *star–star* connections, *cluster–cluster* connections, and *star–cluster* connections (see *Methods*). We observed that bridges were far more likely to connect core cluster hyperedges to peripheral stars than expected by chance ( $P\;<\;1\times 10^{-20}$; Fig. 5B). These results demonstrate that bridge hyperedges are key integrative components in the core–periphery functional architecture of the human brain.

**Figure 6:**
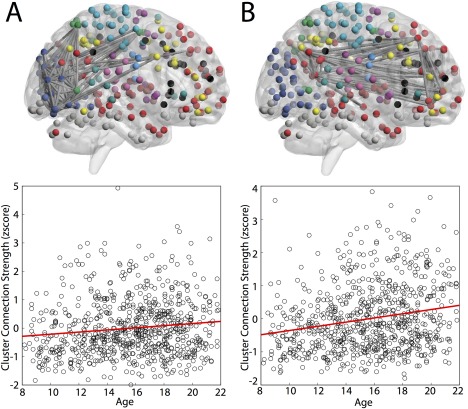
Cluster hyperedges track developmentally driven covariation of functional connections. Two clusters displayed age‐related increased in average strength, as tested by a linear model with age and movement. Panel (A) displays the relationship between edge strength and age in cluster 2, composed predominantly of regions in the visual system: F = 6.34,  P = 0.00185. Panel (B) displays the relationship between edge strength and age in cluster 5, composed predominantly of regions in the default mode: $F\;=\;17.8,\;\;P\;=\;2.69\times 10^{-8}$. [Color figure can be viewed at http://wileyonlinelibrary.com]

### Developmental Effects are Concentrated in Cluster Hyperedges

Having defined the architecture, anatomy, and topological role of each hyperedge archetype, we next examined whether these co‐varying structures were specifically driven by development‐related changes in brain connectivity. To address this question, we tested whether different hyperedge archetypes exhibited differential age‐related differences in strength over the period of adolescence. Specifically, we computed the average edge weight within each hyperedge and measured the correlation between edge weight and age, while covarying for in‐scanner motion. We observed that correlations with age differed by hyperedge class, with clusters displaying the largest increases in strength over age (one‐way ANOVA: F=6.56, df=2, P=0.0016). Post‐hoc comparisons with permutation tests confirmed that stars and bridges displayed weaker correlations with age than clusters (*P* = 0.0019 and *P* = 0.0381, respectively). Next, for each hyperedge in the collection of three archetypes, we calculated the partial correlation between average edge weight and age while controlling for in‐scanner motion. We then applied a correction for multiple testing with FDR q≤0.01 on the associated *P* values. We observed that none of the stars or bridges remained significant; by contrast, two clusters displayed an average strength that was significantly correlated with age (Fig. 4): cluster 2 in the visual system, where the *t*‐tests of the coefficients gave t = 3.25, p = 0.0012, for age, and cluster 5 in the default mode with t = 5.70, $p\;=\;1.69\times 10^{-8}$.

## DISCUSSION

We developed and applied a novel approach to examine high‐resolution edge‐based hypergraphs in the developing human brain. Hypergraphs are composed of hyperedges, which are groups of functional connections whose strengths co‐vary with one another across subjects. We applied this approach to resting state data acquired from 780 youth, uncovering an edge‐based core–periphery structure whereby peripheral stars are linked to clusters in the functional core via topological bridges. Stars were centered on specific brain regions and clusters recapitulated well‐known cognitive systems including visual, default mode, salience, and cingulo‐opercular systems. Clusters in the topological core of the hypergraph were driven by development‐specific changes in resting state brain dynamics. By treating a functional connection as the fundamental unit of interest, these findings suggest a new conceptualization of brain organization that is not offered by typical network analyses.

### Clusters Recapitulate Specific Functional Brain Modules

Cluster hyperedges corresponded to some of the well‐known cognitive systems described in the neuroimaging literature, including the visual, motor, default, and cingulo‐opercular/salience systems. The default mode network was split into two components, both of which included connections with the central regions of the ventromedial prefrontal cortex and posterior cingulate/precuneus. Notably, one default mode hyperedge was preferentially focused around the medial temporal lobe, paralleling prior accounts of default‐mode subsystems [Andrews‐Hanna et al., 2010].

Beyond their anatomical specificity, clusters are also topologically poised to perform specific functions. Indeed, clusters are enriched for highly connected hub edges (Supporting Information, Fig. S1), and therefore form a relatively stable basis around which all other functional associations in the brain can evolve over development. Such edge cores are conceptually similar to core regions in the brain's “rich club” [Van Den Heuvel and Sporns, 2011], and may similarly mediate information transfer over integrating connections involving sensorimotor processes and the default mode network. Clusters are thus well‐situated to serve as the backbone of developing information processing capabilities throughout adolescence.

### Stars: Local Motifs Distributed Across the Brain

While cluster hyperedges align well with a few of the known large‐scale cognitive systems from previous regionally based network analyses, our high‐resolution edge‐based network analysis additionally uncovered a novel subnetwork type that we refer to as the star hyperedge. Stars are centered around a small number of brain regions (one, two, or three), with edges that radiate outward. Notably, such star‐shaped systems cannot be detected by network analyses where nodes are represented by regions rather than edges: community detection techniques by definition will group together regions with similar patterns of connectivity [Bassett et al., 2011b]. Indeed, when community detection analyses of regionally based networks identify a subnetwork with a single node, such a result is generally considered a fragment and not considered further [Bassett et al., 2013a].

Intuitively, star‐shaped subnetworks may represent key partitions of connections that integrate processes from diverse sources within a single node or broadcast to other nodes. Such a regionally focused account of specialized functional networks is consistent with lesion‐based data from both animals and humans, where localized injury may have highly specific functional consequences [Langlois et al., 2006; Alstott et al., 2009]. In contrast to the densely connected core where cluster hyperedges are concentrated, stars are present in the relatively sparsely connected hypergraph periphery. These peripheral stars tend to be found in the frontal–parietal systems and other multimodal regions [Sepulcre et al., 2012] that play central roles in intramodule and intermodule communication.

### Bridges Link Stars to Clusters

In addition to star‐shaped formations, the hypergraph approach identified the presence of bridge hyperedges for the first time. In contrast to clusters, which have locally dense connections, bridges exclusively link two disparate sets of brain regions. We found that bridges preferentially linked clusters in the brain's functional core to stars in the periphery. The linking architecture of a bridge hyperedge implies a critical role within the brain's core–periphery framework, potentially facilitating information flow between highly segregated functional systems and regions where distributed higher order processing occurs. As with the stars, bridges cannot be identified using typical regional‐based network analysis. It is important to note that we did not predict the discovery of these bridges. However, bridge‐like (or bipartite) formations are a frequent feature of other types of systems, including microbial complexity networks [Corel et al., 2016] and microbiome data [Sedlar et al., 2016].

### Development Drives Co‐Varying Functional Connections

As a final step, we examined how hypergraphs developed during youth. Developmental associations were concentrated among cluster hyperedges, suggesting that the functional architecture captured by stars and bridges may develop earlier in life and be stable during the late childhood and adolescent epochs. Specifically, we found significant associations with age in the strength of the default mode and visual hyperedges. These results accord with a pattern of network segregation: the visual and default mode systems have very strong within‐network connectivity, and relatively limited connectivity between other brain networks [Power et al., 2011]. Their strengthening during development is consistent with a pattern of network segregation [Gu et al., 2015b, Satterthwaite et al., 2013] that could support a greater diversity of the brain's dynamic repertoire [Betzel et al., 2016] and an enhanced capability for adaptation [Mattar et al., 2016].

It is important to consider these finding in light of current literature on developmental changes in functional connectivity over similar age ranges [Menon, 2013; Di Martino et al., 2014]. For example, in 82 subjects from the ages of 8–24 years, Chai et al. [2014] demonstrate that the default mode system becomes increasingly segregated from task‐positive systems during development. In 99 subjects from the ages of 10–20 years, Hwang et al. [2013] studied the developmental emergence and stability of hubs in resting‐state functional brain networks, demonstrating that while hubs were present in late childhood, the connectivity between hubs and nonhubs continues to change into young adulthood, potentially supporting mature cognitive function. In 192 subjects from the ages of 10–26 years, Marek et al. [2015] confirm the broad notion that networks stabilize prior to adolescence and subsequently modulate their integration to support cognitive performance. Our study complements these previous efforts by examining not simply the average functional connectivity within or between known cognitive systems, but also by identifying and characterizing a data‐driven group of edges that co‐vary in their strength over 780 subjects.

### Methodological Considerations

Some limitations apply to this study. While the parcellation was selected for its robustness and prominence in the literature [Power et al., 2011], other schemes are available and may offer additional insights [Bassett et al., 2011a]. In particular, this atlas undersamples subcortical and cerebellar regions, which may be particularly important in development. In addition, while the edge‐by‐edge hypergraph representation is applicable to all estimates of functional connectivity [Bassett et al., 2014], we employed a pairwise coherence between region time series [Zhang et al., 2016]. Furthermore, inference regarding developmental effects is limited by the use of a cross‐sectional dataset; longitudinal research designs would be a useful complement to corroborate the findings reported here. Finally, it will be interesting in future to better understand the relationship between hyperedge strength and individual differences in behavior, a question that would benefit from multivariate statistical approaches including partial least squares [Krishnan et al., 2011] and canonical correlation analysis [Bruguier et al., 2008].

## AUTHOR CONTRIBUTIONS

TDS and DSB designed research; SG, MZ, TDS, and DSB performed research; SG and DSB contributed new reagents/analytic tools; SG, MY, TDS, and DSB analyzed data; and SG, MZ, TDS, JDM, REG, RCG, and DSB wrote the article.

## Supporting information

Supporting Information
