# Supplementary material for: Functional hypergraph uncovers novel covariant structures over neurodevelopment
Source: Hum Brain Mapp. 2017 May 11;38(8):3823–35. doi: 10.1002/hbm.23631 (PMC6323637; doi:10.1002/hbm.23631)
Supplement: Supplementary file 1 — Supporting Information [file HBM-38-3823-s001.pdf]

Supplement Information for "Functional Hypergraph Uncovers Novel Covariant Structures over Neurodevelopment"

Shi Gu[a,d,e,†], **Muzhi Yang**[a,b,e,†], **John D. Medaglia**[c], **Ruber C. Gur**[a], **Raquel E. Gur**[a], **Theodore D. Satterthwaite** [a,‡], **and Danielle S. Bassett**[d,e,*,‡]

[a]Department of Psychiatry, University of Pennsylvania, Philadelphia, PA 19104, USA; [b]Applied Mathematics and Computational Science Graduate Group, University of Pennsylvania, Philadelphia, PA 19104, USA; [c]Moss Rehabilitation Research Institute, Elkins Park, PA 19027; [d]Department of Electrical and Systems Engineering, University of Pennsylvania, Philadelphia, PA 19104, USA; [e]Department of Bioengineering, University of Pennsylvania, Philadelphia, PA; [†, ‡]Contributed equally to this work.

Supplementary Results

Clusters are enriched for hubs. To describe the topological role of each type of hyperedge, we first identified *hub* edges within the hypergraph. We defined the *strength* of an edge m to be the mth column sum of the hypergraph $\mathbf{H}$, and we defined a *hub edge* as an edge that lay in the top 1% of strengths. We observed that hub edges are predominantly located within cluster hyperedges (Fig. S1A). In contrast, stars contain the fewest hub edges, with bridges containing an intermediate number (Fig. S1B). Note that the number of hub edges within each type is proportional to the total number of edges in that type, after subtracting that expected in the null distribution. This differential distribution of hub edges throughout the three types suggests that clusters form a densely connected functional core within the hypergraph, while less-connected stars occupy the hypergraph's periphery. Furthermore, it raises the possibility that the bridge hypergraphs may explicitly link densely connected core clusters and peripheral stars. The post-hoc multiple comparisons with FDR $q < 0.001$ reject all the null hypothesis of no difference among groups.

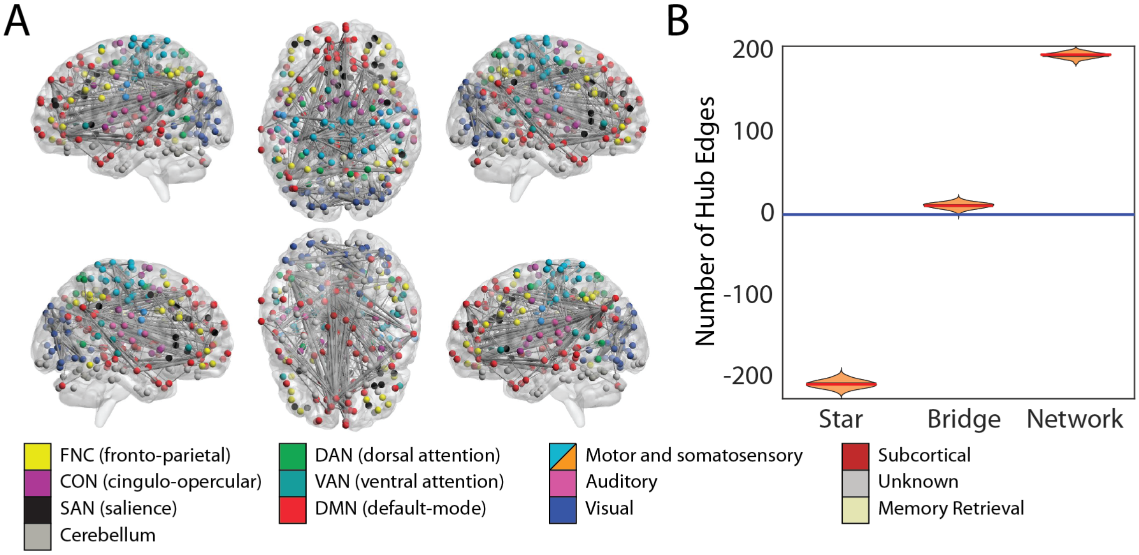

Fig. S1. Anatomical Distribution of Hub Edges. *(A)* We observed that hub edges – functional connections that strongly co-evolve with one another – are predominantly located in visual, motor, and default mode areas. *(B)* Hub edges are more likely to concentrate in the cluster hyperedges than in stars or bridges.

Architecture of the hypergraph with age, sex, and movement regressed. To determine whether our results were biased by the age, sex, and movement of participants, we constructed a separate hypergraph after regressing out participant age, sex, and movement from each edge in the network. Next, we performed the clustering with the same parameter settings as those reported in the main text, and we tested whether bridges were more likely to connect stars with clusters. We found that bridges were more likely to connect stars with clusters than expected under the null hypothesis ($p < 10^{-20}$) and less likely to connect stars to other stars than expected under the null hypothesis ($p < 10^{-20}$); see Figure. S2.

Supplementary Methods

Subjects. The PNC is a collaboration between the Center for Applied Genomics at Children's Hospital of Philadelphia (CHOP) and the Brain Behavior Laboratory at the University of Pennsylvania (Penn). Study procedures were approved by the

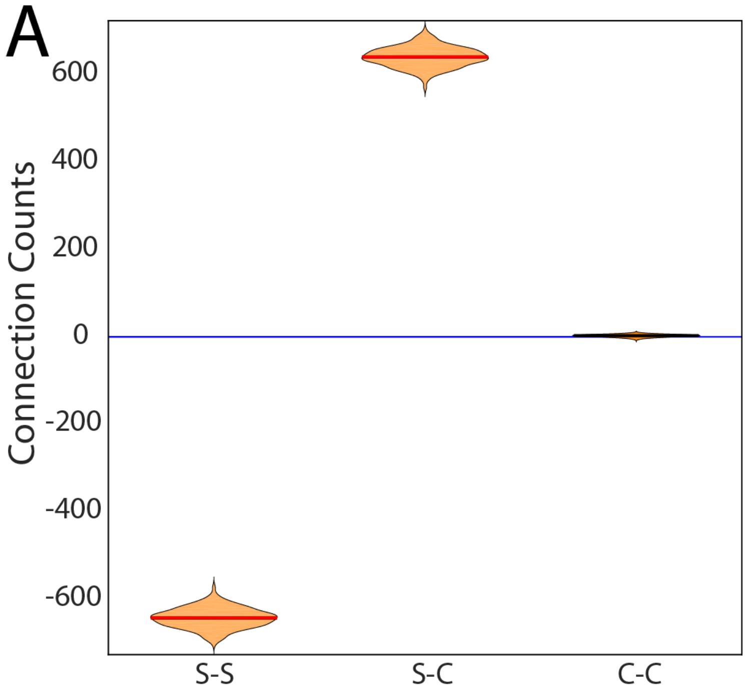

Fig. S2. Bridges Connect Stars to Clusters. *(A)*Testing the intersection of hyperedges versus the null distribution where the nodal occupation is uniformly sampled from all brain regions (see Methods), we demonstrate that bridges are more likely to connect stars to clusters than expected ($p < 10^{-20}$). Moreover, bridges were less likely to connect stars to other stars ($p < 10^{-20}$).

Institutional Review Boards of both Penn and CHOP. The target population-based sample is of 10,000 youths who presented to the CHOP network for a pediatric visit and volunteered to participate in genomic studies of complex pediatric disorders [1]. A subsample of 1,445 participants, stratified by age and sex, were randomly selected for neuroimaging [2]. Of these, 1,275 had resting-state data acquired. Participants were excluded due to missing cognitive data, poor imaging data quality, or a history that suggested potential abnormalities of brain development. Data regarding medical history was gathered from both self-report at time of study entry as well as electronic medical records available from CHOP. Specifically, 234 participants were excluded due to a history of medical problems that might affect brain function, a history of inpatient psychiatric hospitalization, or current use of psychotropic medication. Additionally, 323 participants met exclusion criteria due to poor resting-state image quality [3–5], including if scan mean relative displacement exceeded 0.2mm (see below), if there were greater than 20 volumes with relative displacement greater than 0.25mm, or if gross between-run motion resulted in incomplete brain coverage. These exclusion criteria resulted in a final eligible pool of 780 participants aged 8–22 years (mean age 15.63 (SD=3.28); 333 males; see Figure S3 for the distribution of ages in our sample). Many participants were excluded due to multiple criteria.

Time series preprocessing. All functional timeseries were slice-time corrected, motion corrected to the median image using a tri-linear interpolation with six degrees of freedom [6], and spatially smoothed (6mm FWHM, isotropic). Confound regression was performed with a 36-parameter model [7, 8] including 6 motion parameters and the temporal derivative, quadratic term, and temporal derivative of the quadratic of each and global signal regression [3, 4]. Before confound regression, the confound parameters were band-pass filtered identically to the timeseries to prevent mismatch in the frequency domain and to permit confound parameters to best fit retained signal frequencies [9]. Because spike regression and scrubbing have been found to bias graph properties, they were not applied [8]. Processed subject-level BOLD images were coregistered to the subject's T1 image with boundary-based registration with integrated distortion correction from FSL 5 [10]. Whole-head T1 images were registered to the Montreal Neurological Institute 152 1mm template with top-performing diffeomorphic SyN registration from ANTs [11–13]. Each subject's registration was inspected manually and evaluated for accuracy using spatial correlations. Network nodes were registered to subject space for timeseries extraction by concatenating the coregistration, distortion correction, and normalization transformations such that only one interpolation was performed in the entire process.

Hyperedges: Clusters of co-evolving functional connections. We hypothesized that functional connections would vary with one another over subjects, being driven by collective neurophysiological processes from intrinsic computations, extrinsic stimulation, or mutually trophic effects on connectivity [14]. To test this hypothesis, we sought to identify significant clusters of co-evolving

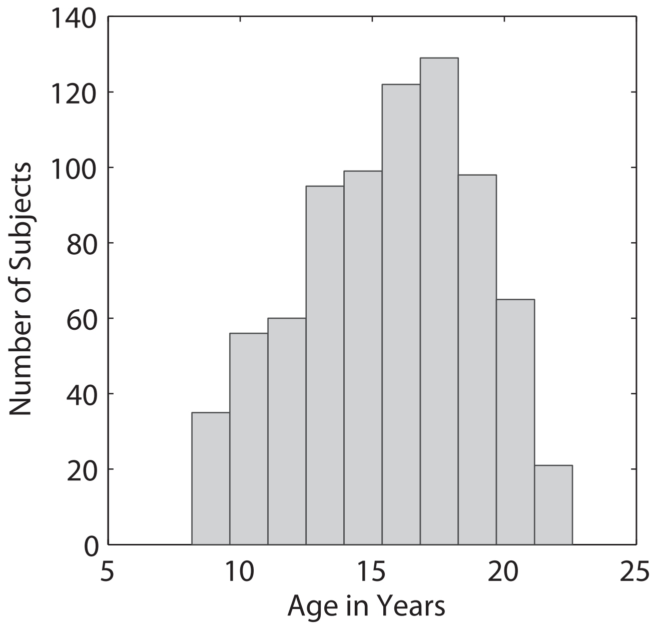

Fig. S3. Distribution of Ages in PNC Cohort A histogram showing the number of subjects from ages 8 to 22 years that were included in this study. N=780 participants (mean age 15.63 (SD=3.28); 333 males).

edges by applying a common network-based community detection algorithm [15, 16] to the hypergraph $\mathbf{H}$. Due to its simplicity, computational power, and previous utility in neuroimaging contexts, we chose the modularity maximization technique [17]. Using a Louvain-like locally greedy algorithm [18, 19] we optimized the modularity function

$$Q = \sum_{mn}\left[\left(H^+_{mn} - \gamma^+ \frac{p^+_m p^+_n}{2\mu^+}\right) + \left(H^-_{mn} - \gamma^- \frac{p^-_m p^-_n}{2\mu^-}\right)\right]\delta(c_m, c_n) \quad [1]$$

where H^+ is the positive part of $\mathbf{H}$ and H^- is the negative part of $\mathbf{H}$, $p^+_m = \sum_n H^+_{mn}$, $p^-_m = \sum_n H^-_{mn}$, $\mu^+ = \sum_{mn} H^+_{mn}$, $\mu^- = \sum_{mn} H^-_{mn}$, γ^+ and γ^- are resolution parameters that tune the relative size of clusters detected. After performing a fine-scale parameter sweep, we observed that $\gamma^+ = \gamma^- = 7$ offered the greatest stability in cluster structure (See supplementary results below). We refer to each cluster in the hypergraph $\mathbf{H}$ as a hyperedge, since it represents a collection of functional connections (edges) that co-evolve with one another [20].

Next, we wished to determine the statistical significance of each cluster, which we obtained by nonparametric permutation testing. We began by denoting $G = (V, H)$ to be the network of E edges, where $V = \{1, \cdots, E\}$ is the edge set, $H \in \mathbb{R}^{E\times E}$ is the adjacency matrix that represents the hypergraph, and H_{mn} is the weight of the link between edge m and edge n. A community structure is characterized by a partition $\mathcal{C} = \{C_1, \cdots, C_K\}$, where $C_r \subset V$ consists of the edges in the rth community and K is the number of communities in G. Using Eq.1, we define the community-specific modularity, which measures the strength of the rth community [21]. We then generate a null community structure $\mathcal{C}'$, which has exactly the same number of communities and the same number of edges in each community as the original community structure $\mathcal{C}$. To achieve this, we simply permute the order of nodes in V. In this way, we generate $T = 1000$ null community structures, and we then determine their community-specific modularity. The p-value for the significance of a community r is then given by the number of communities r' in the null community structure that display a greater community-specific modularity than the true community r, divided by the number of permutations T (for further details, see final section below). Using this approach, we observed that 363 hyperedges were statistically significant, and we discarded all hyperedges that were not significant.

Hyperedge archetypes. For the significant hyperedges, we defined the hyperedge archetypes that each displayed based on their interpretable spatial configurations in the brain, and their differential contributions to the ongoing dynamic processes. For each hyperedge, we listed the edges that composed the associated community r, and then determined the set of nodes (brain regions) that were touched by at least one of those edges. We then defined a binary adjacency matrix $\mathbf{B^r}$ whose elements B^r_{ij} indicated the presence (1) or absence (0) of an edge between nodes i and j that were also present in the community r. We observed that the matrices $\mathbf{B^r}$ fell into one of three categories: focal star hyperedge, bridging bipartite hyperedge, and cluster hyperedge (see Fig. 2 in the main text). We more simply refer to them as *star*, *bridge* and *cluster* hypergraphs. Stars consist of edges that are linked to one another via one, two, or three nodes. Bridges consist of edges that connect one set of nodes to a

second set of nodes, but do not connect nodes within the same set. Clusters consist of edges that connect nodes both within and between sets.

To classify hyperedge types directly from the data, we implemented a hierarchical approach. First, we applied a bipartivity test to the matrix $\mathbf{B^r}$ to determine whether the rth hyperedge was a bipartite structure or a network structure [22]. If the matrix failed the bipartivity test, we assigned the matrix to the *cluster* archetype. If the matrix passed the bipartivity test, we calculated its degree distribution to determine whether it was indeed a *bridge* or better described as a *star*. Specifically, if a matrix was found to involve one, two or three nodes with two or more edges and each other node was associated with exactly one of these three nodes, we assigned the matrix to the *star* archetype; we assigned the matrix to the *bridge* archetype otherwise.

Choices of resolution parameter in modularity maximization. As described earlier in this supplementary methods section, we applied common community detection method to identify hyperedges within the hypergraph. The GenLouvain algorithm that we used is essentially a greedy algorithm that maximizes the modularity quality function at different values of the structural resolution parameter γ. Lower values of γ lead to a few large hyperedges while larger values of γ lead to more smaller hyperedges. In the main text, we chose $\gamma = 7$ because it provided the most stable community architecture across multiple optimizations of the modularity quality function. Here we describe the data supporting that choice. Specifically, we run the GenLouvain algorithm 50 times to get 50 partitions for each different γ ranging from 1 to 10. For each fixed γ, we calculated the z-score of the Rand coefficient [23] between all pairs of 50 partitions. Note that the z-score of the Rand coefficient is a quantity that measures the similarity of two partitions; the higher the value of z, the more consistent the partitions are over optimizations, and therefore the better tuned the γ is to the underlying community structure present in the data [24]. We observed that the highest z values were present at $\gamma = 7$, which implies that when $\gamma = 7$, the partitions GenLouvain obtained are most similar. The average z *versus* γ plot is shown in Fig. S4.

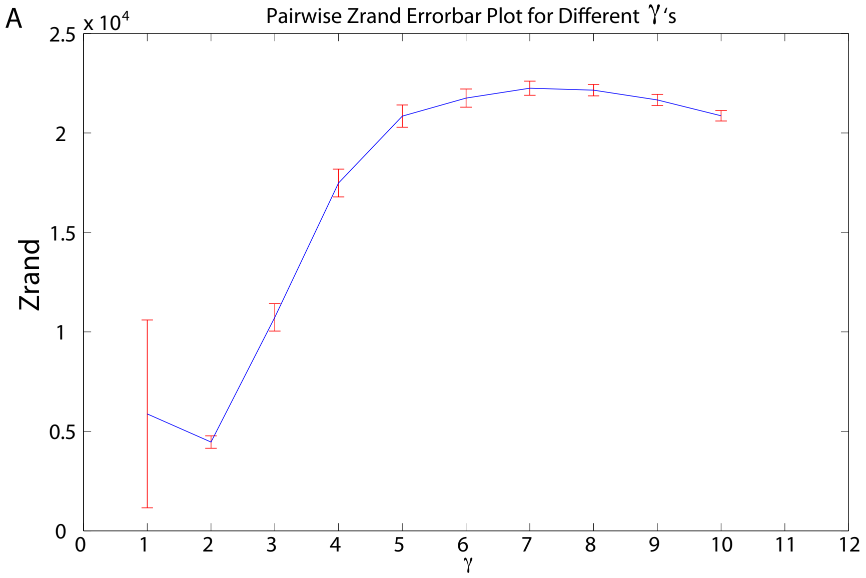

Fig. S4. Choosing the optimal resolution of the community detection. The average z-score of the Rand coefficient as a function of the structural resolution parameter γ in the modularity quality function. Error bars indicated standard error of the mean over optimizations.

Once we obtained the community structure $\mathcal{C}$, we needed to determine which communities were significant. Here we applied a non-parametric significance test (see below) based on permutation to evaluate the significance of each community in the community structure $\mathcal{C}$. Most communities were significant except for a few particularly small communities. We discarded insignificant communities and studied the remaining 364 significant communities (or hyperedges).

Permutation Test for Community Significance. Given a community structure $\mathcal{C}$, its clusterwise-modularity of each cluster in the community structure is defined as

$$Q(C_k|\mathcal{C}) = \sum_{i,j \in C_k} (a_{ij} - \gamma p_{ij}) \qquad [2]$$

, where γ is a resolution parameter, P is a null model adjacency matrix. The modularity $Q(C_k|\mathcal{C})$ measures how strong the kth cluster is, but the problem is that this quantity is not normalized, challenging interpretation. We therefore introduce a permutation test that begins by generating random community structure $\mathcal{C}^r$, which has exactly the same number of clusters and the same number of nodes in each corresponding cluster as in $\mathcal{C}$. To achieve this, we just simply permuted the order of

nodes in V. In this way, we generated T copies of independent of random community structures. Then we calculate $Q(C_k|\mathcal{C}^r)$ for every random community structure $\mathcal{C}^r$. Now define

$$\mathrm{Sig}(C_k|\mathcal{C}) = \frac{Q(C_k|\mathcal{C}) - Q_{max}(k)}{Q_{max}(k) - Q_{min}(k)} \quad [3]$$

, where $Q_{min}(k) = \min_i Q(C_k^{r(i)}|\mathcal{C}^{r(i)})$ and $Q_{max}(k) = \max_i Q(C_k^{r(i)}|\mathcal{C}^{r(i)})$. $\mathrm{Sig}(C_k|\mathcal{C})$ is a normalized quantity that measures the significance of each cluster in a community structure.

References

1. C GR et al. (2012) Age group and sex differences in performance on a computerized neurocognitive battery in children age 8-21. *Neuropsychology* 26:251–265.
2. Satterthwaite TD et al. (2014) Neuroimaging of the philadelphia neurodevelopmental cohort. *Neuroimage* 86:544–553.
3. Satterthwaite TD et al. (2013) An improved framework for confound regression and filtering for control of motion artifact in the preprocessing of restingstate functional connectivity data. *Neuroimage* 64:240–256.
4. Satterthwaite TD et al. (2013) Heterogeneous impact of motion on fundamental patterns of developmental changes in functional connectivity during youth. *Neuroimage* 83C:45–57.
5. Satterthwaite TD et al. (2012) Impact of in-scanner head motion on multiple measures of functional connectivity: Relevance for studies of neurodevelopment in youth. *Neuroimage* 60(1):623–632.
6. Jenkinson M, Bannister P, Brady M, Smith S (2002) Improved optimization for the robust and accurate linear registration and motion correction of brain images. *Neuroimage* 17:825–841.
7. Friston KJ, Williams S, Howard R, Frackowiak RS, Turner R (1996) Movement-related effects in fMRI time-series. *Magn Reson Med* 35(3):346–355.
8. Yan CG et al. (2013) A comprehensive assessment of regional variation in the impact of head micromovements on functional connectomics. *Neuroimage* 76C:183–201.
9. Ciric R et al. (2016) Benchmarking confound regression strategies for the control of motion artifact in studies of functional connectivity. *arXiv* 1608:03616.
10. Greve DN, Fischl B (2009) Accurate and robust brain image alignment using boundary-based registration. *Neuroimage* 48(1):63–72.
11. Avants BB, Epstein CL, Grossman M, Gee JC (2008) Symmetric diffeomorphic image registration with cross-correlation: evaluating automated labeling of elderly and neurodegenerative brain. *Med Image Anal* 12:26–41.
12. Avants BB et al. (2011) A reproducible evaluation of ANTs similarity metric performance in brain image registration. *Neuroimage* 54:2033–2044.
13. Klein A et al. (2009) Evaluation of 14 nonlinear deformation algorithms applied to human brain MRI registration. *Neuroimage* 46:786–802.
14. Bassett DS et al. (2008) Hierarchical organization of human cortical networks in health and schizophrenia. *J Neurosci* 28(37):9239–9248.
15. Porter MA, Onnela JP, Mucha PJ (2009) Communities in networks. *Notices of the American Mathematical Society* 56(9):1082–1097, 1164–1166.
16. Fortunato S (2010) Community detection in graphs. *Physics Reports* 486:75–174.
17. Newman ME (2006) Modularity and community structure in networks. *Proc Natl Acad Sci U S A* 103(23):8577–8582.
18. Blondel VD, Guillaume JL, Lambiotte R, Lefebvre E (2008) Fast unfolding of communities in large networks. *Journal of Statistical Mechanics: Theory and Experiment* 10:P1000.
19. Jutla IS, Jeub LGS, Mucha P (2011–2012) A generalized louvain method for community detection implemented in matlab.
20. Bassett DS, Wymbs NF, Porter MA, Mucha PJ, Grafton ST (2014) Cross-linked structure of network evolution. *Chaos* 24(1):013112.
21. Bassett DS, Owens ET, Porter MA, Manning ML, Daniels KE (2015) Extraction of force-chain network architecture in granular materials using community detection. *Soft Matter* 11(14):2731–2744.
22. Estrada E, Rodriguez-Velazquez JA (2005) Spectral measures of bipartivity in complex networks. *Phys Rev E Stat Nonlin Soft Matter Phys* 72(4 Pt 2):046105.
23. Traud AL, Kelsic ED, Mucha PJ, Porter MA (2011) Comparing community structure to characteristics in online collegiate social networks. *SIAM Review* 53(3):526–543.
24. Bassett DS et al. (2013) Robust detection of dynamic community structure in networks. *Chaos: An Interdisciplinary Journal of Nonlinear Science* 23(1):013142.
